# Impact of ozone exposure on the response to glucocorticoid in a mouse model of asthma: involvements of p38 MAPK and MKP-1

**DOI:** 10.1186/s12931-014-0126-x

**Published:** 2014-10-08

**Authors:** Aihua Bao, Feng Li, Min Zhang, Yuqing Chen, Pengyu Zhang, Xin Zhou

**Affiliations:** Department of Respiratory Medicine, Shanghai First People’s Hospital, Shanghai Jiao tong University, 100 Haining Road, Shanghai, China 200080

**Keywords:** Ozone, Glucocorticoids insensitivity, p38 MAPK, MKP-1, IL-17, Asthma

## Abstract

**Background:**

Molecular mechanisms involved in the oxidative stress induced glucocorticoids insensitivity remain elusive. The mitogen-activated protein kinase phosphatase (MKP) 1 mediates a part of glucocorticoids action and can be modified by exogenous oxidants. Whether oxidant ozone (O_3_) can affect the function of MKP-1 and hence blunt the response to corticotherapy is not clear.

**Methods:**

Here we employed a murine model of asthma established with ovalbumin (OVA) sensitization and challenge to evaluate the influence of O_3_ on the inhibitory effect of dexamethasone on AHR and airway inflammation, and by administration of SB239063, a selective p38 MAPK inhibitor, to explore the underlying involvements of the activation of p38 MAPK and the expression of MKP-1.

**Results:**

Ozone exposure not only aggravated the pulmonary inflammation and AHR, but also decreased the inhibitory effects of dexamethasone, accompanied by the elevated oxidative stress, airway neutrophilia, enhanced phosphorylation of p38 MAPK, and upregulated expression of IL-17. Administration of SB239063 caused significant inhibition of the p38 MAPK phosphorylation, alleviation of the airway neutrophilia, and decrement of the ozone-induced IL-17 expression, and partly restored the ozone-impaired effects of dexamethasone. Ozone exposure not only decreased the protein expression of MKP-1, but also diminished the dexamethasone-mediated induction process of MKP-1 mRNA and protein expression.

**Conclusions:**

The glucocorticoids insensitivity elicited by ozone exposure on current asthma model may involve the enhanced phosphorylation of p38 MAPK and disturbed expression of MKP-1.

**Electronic supplementary material:**

The online version of this article (doi:10.1186/s12931-014-0126-x) contains supplementary material, which is available to authorized users.

## Background

Bronchial asthma is a common airway disease characterized by airway hyperresponsiveness (AHR), airway inflammation, and airway remodeling. Up to date, the most effective treatment of asthma is performed through glucocorticoids (GC), taken systemically or by inhalation. However, up to 10% of the asthmatic patients are clinically insensitive to corticotherapy [1], while the molecular mechanisms contributing to this lack of sensitivity remain unclear. The classical model of the glucocorticoid anti-inflammatory action is established as that the binding of glucocorticoid to its receptor (GR/NR3C1) may transrepress the inflammatory gene transcription or transactivate multiple anti-inflammatory/repressive factors [2]. However, an alternative mechanism of the GC action has gained an increasing body of concern focusing attention on the role of the mitogen-activated protein kinase phosphatase 1 (MKP-1), a phosphatase, induced by glucocorticoids, which inactivates the mitogen-activated protein kinase (MAPKs) [3,4]. It was reported that the genetic inhibition of MKP-1 aggravated the inflammation of an allergic animal model [5] and attenuated the suppressive effect of dexamethasone (Dex) on several pro-inflammatory gene expressions in macrophages [6]. Previously, we observed that the inhibitory effect of dexamethasone on the acetylcholine-induced airway smooth muscle contraction was dependent on the function of MKP-1 [7]. Maneechotesuwan et al. found that the inhibitory effect of fluticasone on the Th2 cytokines production in cultured human T lymphocytes in vitro was mediated by its induction of MKP-1 expression, which further suppressed the p38 MAPK activation and the GATA-3 nuclear translocation [8]. It was revealed by Furst et al. that the intracellular reactive oxygen species (ROS), which are synthesized under the stimulation of corticosteroids, initiate the induction of the MKP-1 expression by GC, through the c-Jun N-terminal kinase (JNK)-activator protein (AP)-1 pathway [9]. Since the intracellular redox status is vulnerable to the exogenous oxidative stress, it sheds light on the hypothesis that the extrinsic oxidants could influence the inducing process of MKP-1 expression by corticosteroids, subsequently interrupting the anti-inflammatory actions of the drugs.

Mounting evidences support an opinion that oxidative stress plays crucial roles in the pathogenesis of glucocorticoids insensitivity [10], such as causing defective histone acetylation and GR modification, etc. [1]. However, few investigations have been conducted to determine whether the oxidants-mediated disruption of MKP-1 also contributes to the resistance to GCs.

O_3_ is an exogenous oxidant which adversely affects human health by irritating the mucosa and harming the respiratory system [11]; it is particularly deleterious when encountered by the lungs of asthmatics. We have reported that acute ozone exposure on a mouse model of allergic asthma caused the aggravation of the pulmonary inflammation, AHR, and mucus hyperproduction [12]. Using a chronic allergic asthma model, we found that repeated O_3_ exposures reduced the response to systemically administrated dexamethasone of the experimental animals [13]. Furthermore, in a human study researchers reported that transient exposure to ozone rendered the stable asthmatic patients more insensitive to the same dosage of inhaled glucocorticoids, compared with those air-exposed patients [14]. O_3_ is a potent stressor, and the exposure of mice to its influence often causes significant activation of p38 MAPK, which plays a crucial role in the ozone-induced pulmonary inflammation and AHR [15]. Taken into account that the over-phosphorylation of p38 MAPK might be a reflection of impaired dephosphorylation, it is reasonable to infer that ozone might inhibit the function of MKP-1. Generally, we hypothesized that the oxidant ozone might exert some negative effects on the function of MKP-1, and hence interfere with the anti-inflammatory action of GCs.

To verify this hypothesis, we employed a murine asthma model established with ovalbumin (OVA )-induced asthma model mice, in which the animals were exposed to filtered air or 1.0 ppm O_3_ and administered with dexamethasone in combination with or without a selective p38 MAPK inhibitor, SB239063. Their evaluation was performed thereafter, including the observation and estimation of parameters, such as the pulmonary inflammation, AHR, the phosphorylation of p38 MAPK, and the expression of MKP-1. The results of the current study indicated that the inhibitory effect of dexamethasone was compromised after the O_3_ exposure, which was associated with the O_3_-enhanced phosphorylation of p38 MAPK and O_3_-decreased expression of MKP-1 protein.

## Methods

### Animals

Six-week-old female Balb/c mice weighting 18 ~ 20 g were purchased from SLAC Laboratory Animal Co. Ltd.(Shanghai, China) and bred under specific-pathogen-free conditions and kept on an ovalbumin (OVA)-free diet in our own facility.

### Ethics statement

This study was carried out in strict accordance with the recommendations of the Shanghai Committee for Accreditation of Laboratory Animal. The protocol was approved by the Shanghai First People’s Hospital Institutional Review Board (Permit Number: 2010KY047). All surgery was performed under sodium pentobarbital anesthesia, and all efforts were made to minimize suffering.

### OVA sensitization and challenge

The protocol of experimental model establishing was adopted as previously described [12]. Mice were sensitized IP with 20 μg chicken OVA (Grade V, Sigma Aldrich Co., St. Louis, MO) emulsified in 2.0 mg of alum (Shanghai No.4 Reagent & H.V. Chemical Industries Ltd., Shanghai, China) in a total volume of 100 μl of 0.9% sterile saline on days 1 and 14. Non-sensitized mice just received 2.0 mg of alum in 0.9% saline. On day 24, 25, and 26, mice received three aerosol challenges with 5% OVA (wt/vol) (Grade II, Sigma Aldrich Co., St. Louis, MO) in 0.9% endotoxin-free saline (non-sensitized mice received saline only) for 30 min daily. Using a plastic box (50 × 30 × 40 cm^3^) as exposure chamber, where the air was kept at 20 – 22°C and relative humidity 50 – 60%, the aerosol was generated by a sidestream jet nebulizer (PARI BOY, Starnberg, Germany) into the chamber and mice were put into stainless cages inside the chamber with irradiated food and acidified water provided *ad libitum*. One hour before each challenge, mice received dexamethasone (2 mg/kg) IP in 0.1 ml endotoxin-free PBS or PBS alone. The dosage of dexamethasone has been adopted by multiple publications [16].

### Ozone exposure and SB239063 delivery

**Figure 1:**
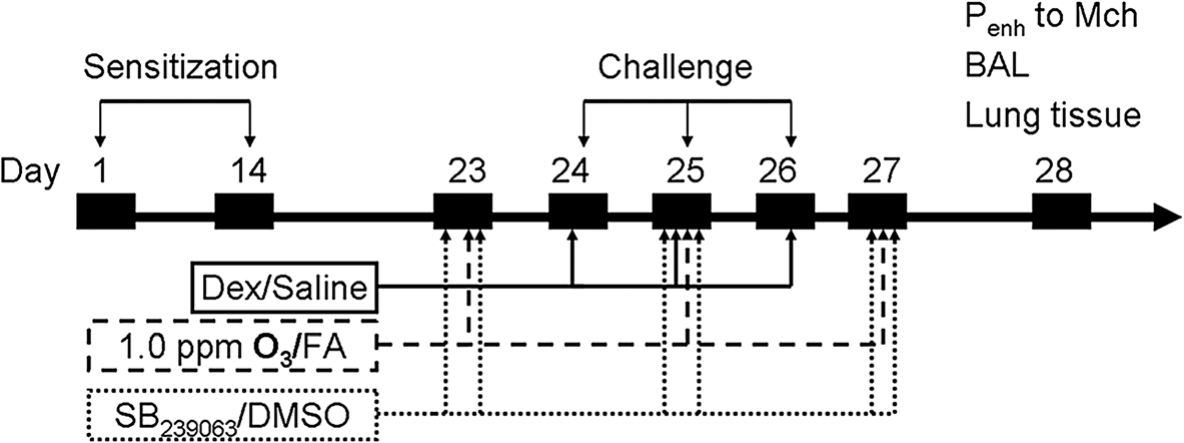
**Schematic diagram of experimental protocol.** Mice were sensitized at day 1 and 14, and challenged at day 24, 25, 26 with OVA or saline. Dexamethasone or saline was administrated intraperitoneally 1 hr before each challenge. Equal number of mice were selected to expose to 1.0 ppm O_3_ or filtered air for 3 consecutive hrs on day 23, 25 and 27. In p38 MAPK inhibition experiment, same amount of animals were injected through tail vein with SB239063 or DMSO. On day 28, measurements were performed including enhanced pause (P_enh_), BAL cells and cytokines, lung tissues for pathological staining and biological testing.

On days 23, 25 and 27, conscious mice were exposed to ozone [1.0 parts/million (ppm) for 3 h] or filtered air. Ozone was generated by directing an air stream with a micro air pump (SC3601PM, Shenzhen, China) into a AQUA MEDIC O_3_ generator (model 300, AB Aqua Medic GmbH, Bissendorf, Germany). Ozone concentration in the chamber was monitored continuously by an Ozone Switch (OS-4, Newark, USA), which also controlled the operation of the ozone generator in response to the instantaneous value. The target O_3_ concentration range was set as 0.77 ppm ~ 1.32 ppm, which has been proved by our preliminary experiment to be able to maintain a comparatively stable O_3_ concentration averaged as 1.01 ± 0.02 ppm (data not shown). This methodology of O_3_ exposure has been adapted by previous study which had documented that, such level of ozone inhalation can induced the airway inflammation but not influence the body weight of mice [17], which means less toxic effect. One hour before and 4 hrs after the O_3_ exposure, mice were injected through tail vein SB239063 (Sigma Aldrich, St. Louis, MO) dissolved in 3% DMSO (0.1 ml, Sigma Aldrich, St. Louis, MO) or DMSO only, respectively. The dosages of SB239063 were titrated in a preliminary experiment, where the final dosage of 4.0 mg/kg was selected. The present experimental protocol was outlined in Figure 1.

### Airway responsiveness testing

Airway responsiveness was assessed noninvasively using a whole-body plethysmography (Buxco., NY, USA) in conscious, unrestrained mice as described previously [18]. Briefly, in day 28, mice were placed in individual chambers. After ten minutes adaptation they were exposed to aerosolized PBS (to establish baseline), followed by increasing concentrations of aerosolized methacholine (Mch, Sigma Aldrich, St. Louis, MO) (1.5625, 3.125, 6.25, 12.5, 25, 50 mg/ml). Each dose of Mch was delivered for 0.5 minute, and respiratory measurements were recorded and averaged over a 7-minute period from the beginning of nebulization. Airflow obstruction was expressed as enhanced pause (P_enh_), which correlates well with airway resistance, and calculated as: P_enh_ = {[Te (expiratory time)/Tr (relaxation time)]-1} × [Pef (peak expiratory flow)/Pif (peak inspiratory flow)]. P_enh_ after each concentration was expressed as percentage change from baseline. The log concentrations of MCh required to increase P_enh_ by 100% from baseline was calculated (LogPC_100_P_enh_).

### Bronchoalveolar lavage and measurements of BALF cytokines

Immediately after the assessment of airway reactivity, mice were sacrificed by an overdose injection of pentobarbital (500 mg/kg i.p). The tracheal was exposed and intubated with PE-60 tubing (0.72-mm inner diameter, 1.22-mm outer diameter). Three aliquots of 0.3 ml sterilized saline were instilled and retrieved as the bronchoalveolar lavage fluid (BALF). Return volume was recorded and was consistently > 60% of the instilled volume. The BALF was then centrifuged at 1000 × g for 10 min at 4°C. The supernatant was aliquoted and stored at −80°C until assay. The remaining cell pellet was resuspended in 1 ml PBS solution. Total cell counts were determined using a haemocytometry, by adding 100 μl of the cell suspension to 100 μl trypan blue stain. Differential cell counts were performed on cytocentrifuge preparations (Cytospin 2; Shandon, UK) stained with Wright-Giemsa by counting 200 cells under × 400 magnification from each individual by the same inspector in a blinded manner. Cells were identified by standard morphology and differentiated into neutrophils, eosinophils, lymphocytes, and macrophages.

Concentrations of soluble mediators in BALF supernatants were determined by enzyme-linked immunosorbent assay (ELISA), as previously described [19]. Measurements of IFN-γ, IL-6 and IL-13 were performed by using commercial kits (R&D Systems China Co. Ltd., Shanghai, China), following the manufacturer’s protocol. The limits of detection were 3 pg/ml for IL-6 and IL-13, and 10 pg/ml for IFN-γ.

### Processing of lung tissues and histological scoring of lung inflammation

After BAL, the left lung lobe was removed and placed in 10% neutral-buffered formalin solution and embedded into paraffin. The remains were microdissected and placed into liquid nitrogen and then kept in −80°C until further process. Four-micrometer paraffin sections were placed onto Fisher PLUS slides. After deparaffinization and rehydration, some paraffin sections were stained with hematoxylin and eosin (H&E), dehydrated, and mounted.

Slides were coded and graded by two independent investigators in a blinded fashion using a reproducible scoring system described elsewhere [20]. A value from 0 to 3 per criterion was adjudged to each tissue section scored. Two criteria were scored to document the pulmonary inflammation: peribronchial inflammation and perivascular inflammation. A value of 0 was adjudged when no inflammation was detectable, a value of 1 for occasional cuffing with inflammatory cells, a value of 2 for most bronchi or vessels surrounded by thin layer (one to five cells) of inflammatory cells and a value of 3 when most bronchi or vessels were surrounded by a thick layer (more than five cells) of inflammatory cells. Total lung inflammation was defined as the average of the peribronchial and perivascular inflammation scores. 2 ~ 3 tissue sections per mouse were scored, and the inflammation scores was expressed as a mean value of 15 ~ 20 sections per group.

### Immunoblotting

Mouse lung tissues were first incubated in ice-cold 1 × cell lysis buffer(Cell signaling technology, Mass., US) plus 1 mM PMSF and a protease inhibitor cocktail (Sigma Aldrich, St. Louis, MO), as well as a serine/threonine phosphatase inhibitor cocktail2 (Sigma-Aldrich, St. Louis, MO4) for 30 minutes and lysed using Fastprep system (MP Biomedicals, Germany). The Homogenates were centrifuged at 15,000 × g for 15 minutes at 4°C and the concentration of proteins in the supernatants fractions was determined by BCA Protein Assay Kit (Pierce, Thermo Scientific Co, US). Equal amounts of protein (75 μg) from blindly selected samples were renumbered and subjected to 10% SDS–PAGE. Separated proteins were transferred to PVDF membranes (iBlot™ Dry Blotting System, Invitrogen) after electrophoresis at 90 V for 7 min. Membranes were blocked with 5% nonfat dry milk dissolved in TBST buffer (10 mM Tris–HCl (pH 7.5), 150 mM NaCl, 0.1% Tween-20) overnight at 4°C. Binding of the primary antibody was detected by infrared dye-conjugated secondary antibodies and Odyssey system (Li-Cor, USA), and the bands were quantified with densitometry. The source of the antibodies and dilutions used were as follows: mouse anti-p38 MAPK (1:5000 dilution), mouse anti-phospho-p38 MAPK (1:5000 dilution) (Cell Signaling Technology, Mass., US), mouse anti-phospho-MKP-1 (1:4000 dilution) and mouse anti-α-actin (1:1000 dilution) (Sigma Aldrich, St. Louis, MO).

### Real-time reverse transcription-polymerase chain reaction

Total RNA was extracted using the TRIzol® Reagent (Invitrogen, Calif. US) according to the manufacturer’s instruction. RNA (2 μg per sample) was used to synthesise single-stranded complimentary DNA (cDNA) using High Capacity cDNA Reverse Transcription Kit (Applied Biosystems, CA, USA) in a PTC-200 Peltier Thermal Cycler (MJ Research, Watertown, Mass., USA). The cDNA generated was used as a template in subsequent real-time PCR analyses. Transcript levels were determined by Applied Biosystems 7500 Real Time PCR System (Applied Biosystems, CA, USA) using SYBR Green PCR Master Mix Reagent (Qiagen, Germany). The sequences of murine forward and reverse primers used are displayed in Table 1 (Devised by Invitrogen Shanghai Ltd. China).

**Table 1:** **Sequences of target genes**

**primer**	**Forward primer**	**Reverse primer**
IL-5	5′-CCATGCAGAGTCCTCAGAAC AA-3′	5′- TTACTGGAA AGGCCCAAG CA-3′
IL-17	5′- CCTGGCGGCTACAGTGAAG-3′	5′- TTTGGACACGCTGAGCTTTG-3′
TNF-α	5′-AGCCGATGGGTTGTACCTTGTC TA-3′	5′-TGAGATAGCAAATCGGCTGACGGT-3′
CXCL1	5′-TGGCTGGGATTCACCTCAAGAACA-3′	5′-TGTGGCTATGACTTCGGTTTGGGT-3′
MKP-1	5′-CTGCCTTGATCAACGTCTCA-3′	5′-ACCCTTCCTCCAGCATTCTT-3′

The thermal cycling program of qPCR was as follows: Step 1, 15 min at 95°C; step 2, 15 s at 95°C; step 3: 30 s at 60°C; step 4: 30 s at 72°C, with step 2 to step 4 repeated for 40 cycles. Relative gene expression was normalized to β-actin. Data are expressed as fold-increase in RNA expression compared with control animals, which are set at a value of 1.

### Measurement of bio-markers of oxidative stress

Malondialdehyde (MDA) level was utilized to quantify the lipid peroxidation in tissues as previously described [21]. Briefly, following lung homogenate preparation, MDA content was based on the reaction of MDA with thiobarbituric acid (Nanjing Jiancheng Bioengineering Institute, Jiangsu, China) at 95°C. Samples were then heated for 1 h at 95°C and centrifuged at 3000 g for 10 min. The absorbance of the supernatant was measured by spectrophotometry at 532 nm using 1,1,3,3-tetramethoxypropane as an external standard. MDA was expressed in micromole per g weight of wet tissue.

Glutathione peroxidase (GSH-Px) activity in lung supernatants was determined spectrophotometrically with minor modification of methods previously described [22]. The rate of GSSG formation was assessed by measuring the reduction of GSH (Nanjing Jiancheng Bioengineering Institute, Jiangsu, China). The unit of GSH-Px activity was defined as μmpl/L reduced GSH · g protein^−1^ · min^−1^.

### Statistical analysis

Data are expressed as mean ± standard error of the mean (SEM). One-way ANOVA was used to determine the statistical significance of differences among groups. *S-N-K* test was used to correct for multiple comparisons when statistical significances were identified in the ANOVA. Comparisons within each group are made by unpaired *t*-test. Criterion for significance was taken to be *p* ≤ 0.05. For statistical analysis, SPSS program (SPSS Inc. Chicago, IL, USA) was used. For statistical charting, GraphPad Prism 5.0 (GraphPad Software Inc., San Diego, CA) was used.

## Results

### Ozone impaired the inhibitory effects of dexamethasone on lung inflammation and AHR

**Figure 2:**
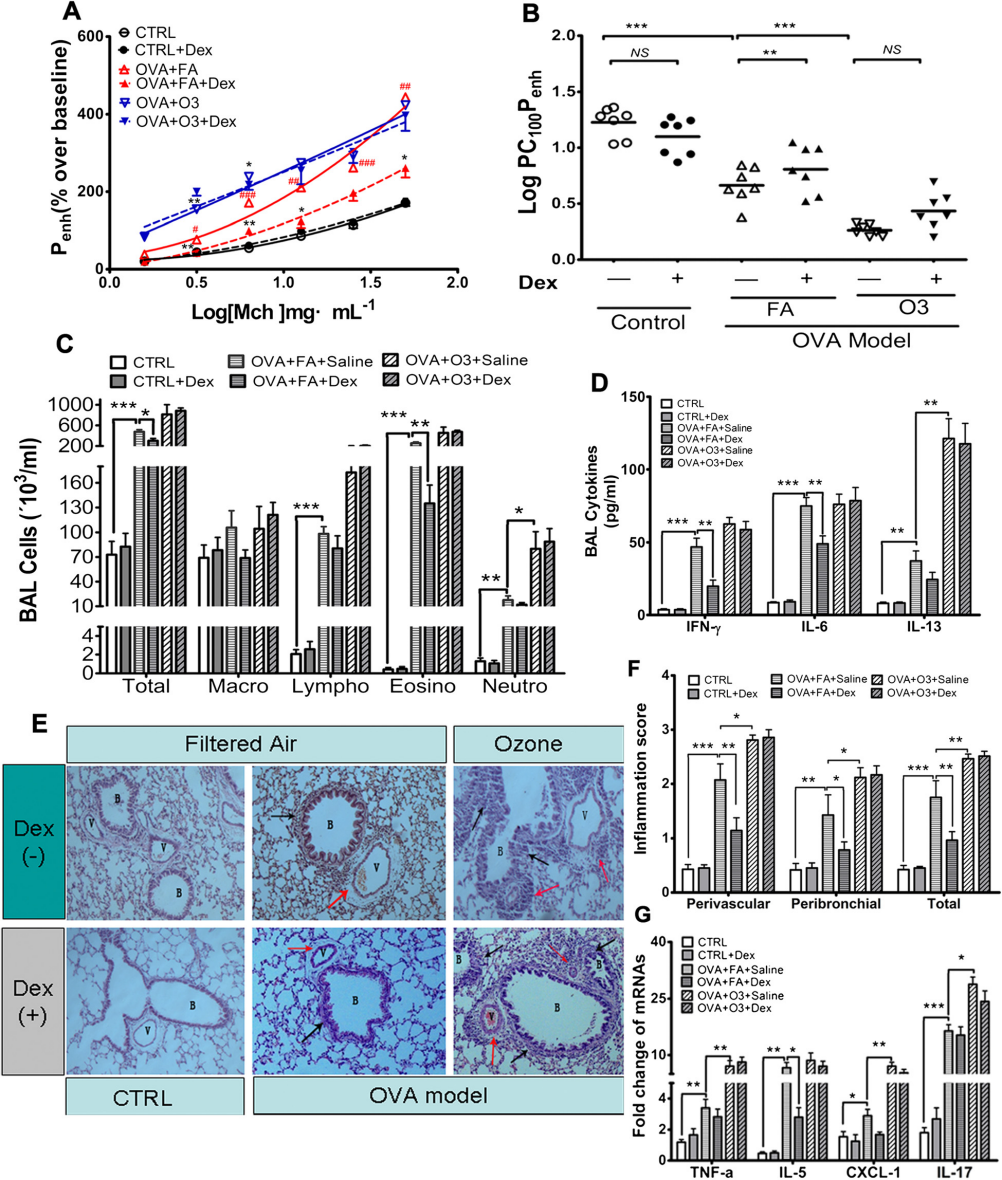
**Ozone inhalation impaired the inhibitory effects of dexamethasone on AHR and lung inflammation.** Twenty-eight mice were sensitized and challenged with OVA and then exposed to 1.0 ppm ozone or filtered air for 3 hrs. Equal amounts of mice in each group (7) were selected to receive intraperitoneal injection either with dexamethasone (2 mg/kg) or with saline only. **A**: Mean percentage increase in enhanced pause (P_enh_) to increasing provocative concentrations of Mch. **B**: log provocative concentration of Mch required to increase P_enh_ from baseline to 100% (LogPC_100_P_enh_). **C**: changes in total cells and differential cellularity of BALF. **D**: BAL cytokines tested by ELISA. **E**: typical changes of haematoxylin staining of lung sections, red arrow: inflammatory cells surrounding vascular; black arrow: inflammatory cells surrounding bronchiole. B: bronchiole, V: vascular. **F**: Inflammation grades in peribronchial and perivascular area of lung seections. **G**: mRNA expression of inflammatory genes. *:p < 0.05; **:p < 0.01; ***: p < 0.001; n = 7.

As expected, compared with the normal controls, mice in the asthma model exhibited AHR (decreased LogPC100Penh), higher BAL number of total cells, lymphocytes, eosinophils and neutrophils, elevated BAL concentration of IFN-γ, IL-6 and IL-13, as well as higher pathological inflammatory score and mRNA expressions of inflammatory cytokines (TNF-α, IL-5, CXCL-1 and IL-17) in lung tissues (Figure 2). Subsequent intraperitoneal administration of dexamethasone (2 mg/kg), but not saline, significantly decreased the AHR, lowered the number of total cells and eosinophils, decreased the BAL concentration of IFN-γ and IL-6, attenuated the pathological inflammation and the mRNA expression of IL-5 in lung tissues (Figure 2).

In comparison with their air-exposed counterparts, O_3_-exposed asthmatic mice had significantly enhanced AHR, increased BAL number of neutrophils and BAL concentration of IL-13, aggravation of lung inflammation, and prominent elevations in lung mRNA expressions of TNF-α, CXCL-1 and IL-17(Figure 2).

Combined administration of dexamethasone on O_3_-exposed asthmatic mice failed to elicit any significant variation on AHR or pulmonary inflammation (Figure 2).

### Ozone increased oxidative stress and p38 MAPK phosphorylation and differentially influenced MKP-1 gene and protein expression

**Figure 3:**
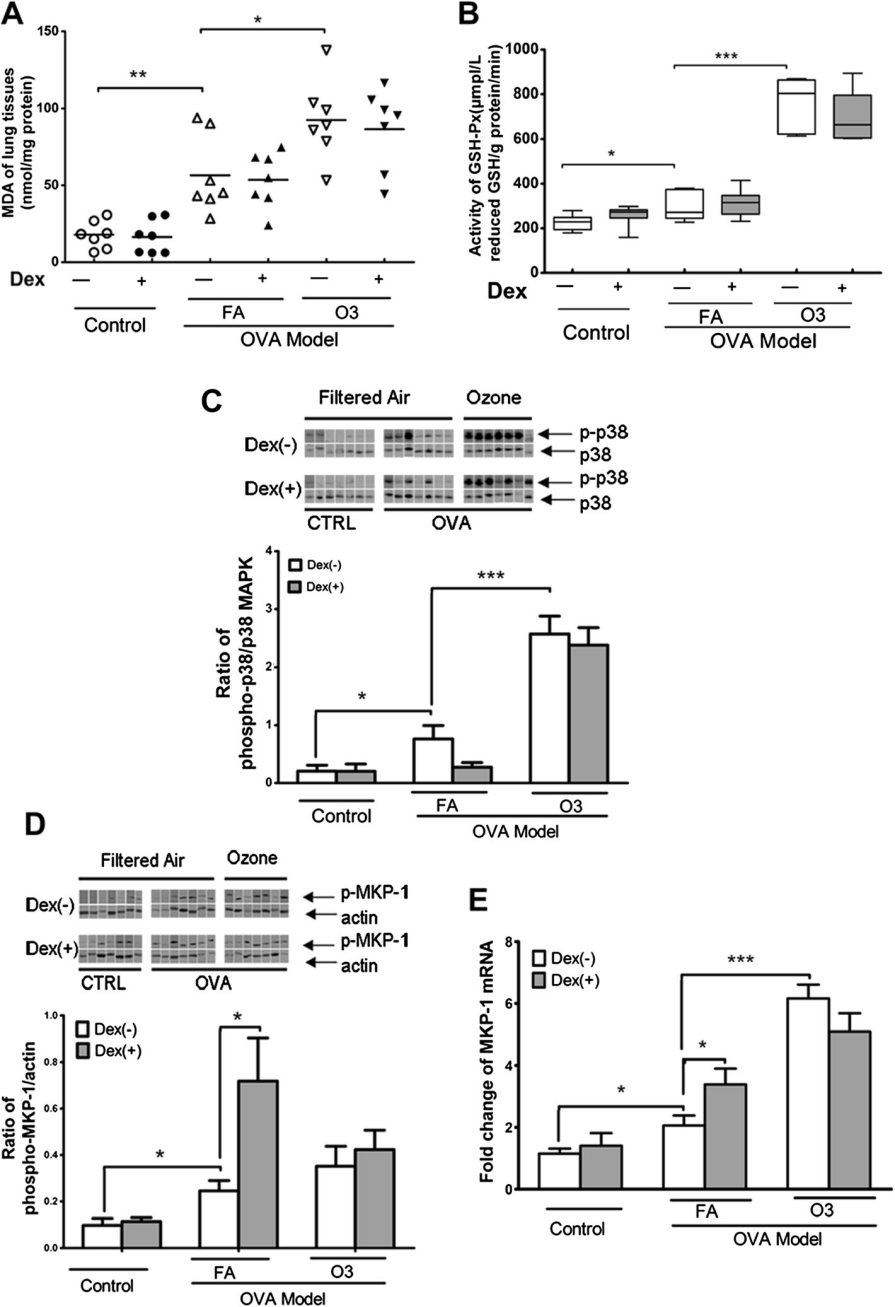
**Ozone induced oxidative stress and changes of phosphorylation of p38 MAPK and expression of MKP-1 in lung tissues. A**: Malondialdehyde (MDA) concentration in lung tissues supernatants measured spectrophotometrically. **B**: Activity of Glutathione peroxidase (GSH-Px) in lung tissue supernatants measured by spectrophotometry. **C**: Western blot analysis of phosphorylated and non-phosphorylated p38 MAPK. The activation of p38 MAPK was determined by the ratio between phospho- and non-phospho- p38 MAPK. The bands were cut from the film where the samples were blindly subjected to polyacrylamide gel electrophoresis and rearranged accordingly. **D**: Western blot analysis of phosphorylated MKP-1 (p-MKP-1) in lung tissues. This was determined as a ratio against β-actin. Band densities were determined and results plotted graphically for the 7 animals in each group. **E**: Fold change of MKP-1 mRNA in lung tissues tested by quantitative RT-PCR (n = 7). *:p < 0.05; **:p < 0.01; ***: p < 0.001.

As a potent exotic oxidant, O_3_ might exert influence on the intrinsic oxidative metabolism. We measured the MDA concentration and the GSH-Px activity in lung tissues and found that the asthmatic mice exhibited higher level of MDA concentration and lower extent of GSH-Px activity than their normal controls. Ozone exposure further significantly intensified the trends of both biomarkers. Further administration of dexamethasone changed neither of them (Figure 3A and B).

To find out whether the p38 MAPK and its phosphatase, MKP-1, get involved in the O_3_ interference with the dexamethasone effect, we evaluated the phosphorylation ratio of the p38 MAPK and the expression of MKP-1. Data showed that the activation of p38 MAPK was prominent in asthma model, in contrast with their normal controls. Ozone exposure on asthma model further enhanced the phosphorylation of p38 MAPK. Administration of dexamethasone on these model animals slightly decreased the phosphorylation ratio of p38 MAPK (saline 0.762 ± 0.232 vs dexamethasone 0.273 ± 0.081, *p* = 0.070). This trend was obscured when the experimental animals received the O_3_ exposure simultaneously (saline 2.571 ± 0.308 vs dexamethasone 2.378 ± 0.302, *p* = 0.663) (Figure 3C).

To evaluate the MKP-1 protein expression level, we measured the phosphorylated protein of this phosphatase, for the phosphorylation promotes its stability and otherwise it is quickly degraded and hence transient [23,24]. Our data showed that both the protein and the mRNA expression of MKP-1 were elevated in allergic mice exposed to air, while the identical group of mice exposed to O_3_ exhibited higher expression of MKP-1 at mRNA level but not at protein level. Administration of dexamethasone on the air-exposed experimental animals increased the expression of MKP-1 at both levels, while this effect was diminished when these asthmatic mice received combined ozone inhalation (Figure 3D and E).

### SB239063 restored partly the ozone-impaired effects of dexamethasone

To further demonstrate the involvements of p38 MAPK in the ozone-induced inhibition of glucocorticoids effects, an inhibitor of p38 MAPK, SB239063, was administrated in mice before and after each O_3_ exposure. The dosage of SB239063 were titrated in a preliminary experiment, in which three dosages of SB239063 (0.4 mg/kg, 1.2 mg/kg, 4 mg/kg; diluted with DMSO) and DMSO alone were randomly injected through tail vein in mice exposed 1.0 ppm ozone for 3 hours (usage referred to the previous study [25]). Some measurements were performed including BAL cells and AHR to evaluate the dose-dependent inhibitory effects of SB239063. The data showed that, compared with DMSO alone and other two dosages, 4 mg/kg SB239063 significantly decreased the number of BAL total cells (O_3_: 400.00 ± 75.86 vs O_3_ + SB239063_4mg/kg_: 170.00 ± 24.06, *p* = 0.028) and macrophages (O_3_: 367.41 ± 69.22 vs O_3_ + SB239063_4mg/kg_: 156.44 ± 46.40, *p* = 0.028), and slightly inhibited the ozone-induced AHR (O_3_: 0.315 ± 0.118 vs O_3_ + SB239063_4mg/kg_: 0.696 ± 0.118, *p* = 0.062) (Additional file 1: Figure S1.). Accordingly, 4 mg/kg was adopted as the dosage of SB239063 during the subsequent experiment.

**Figure 4:**
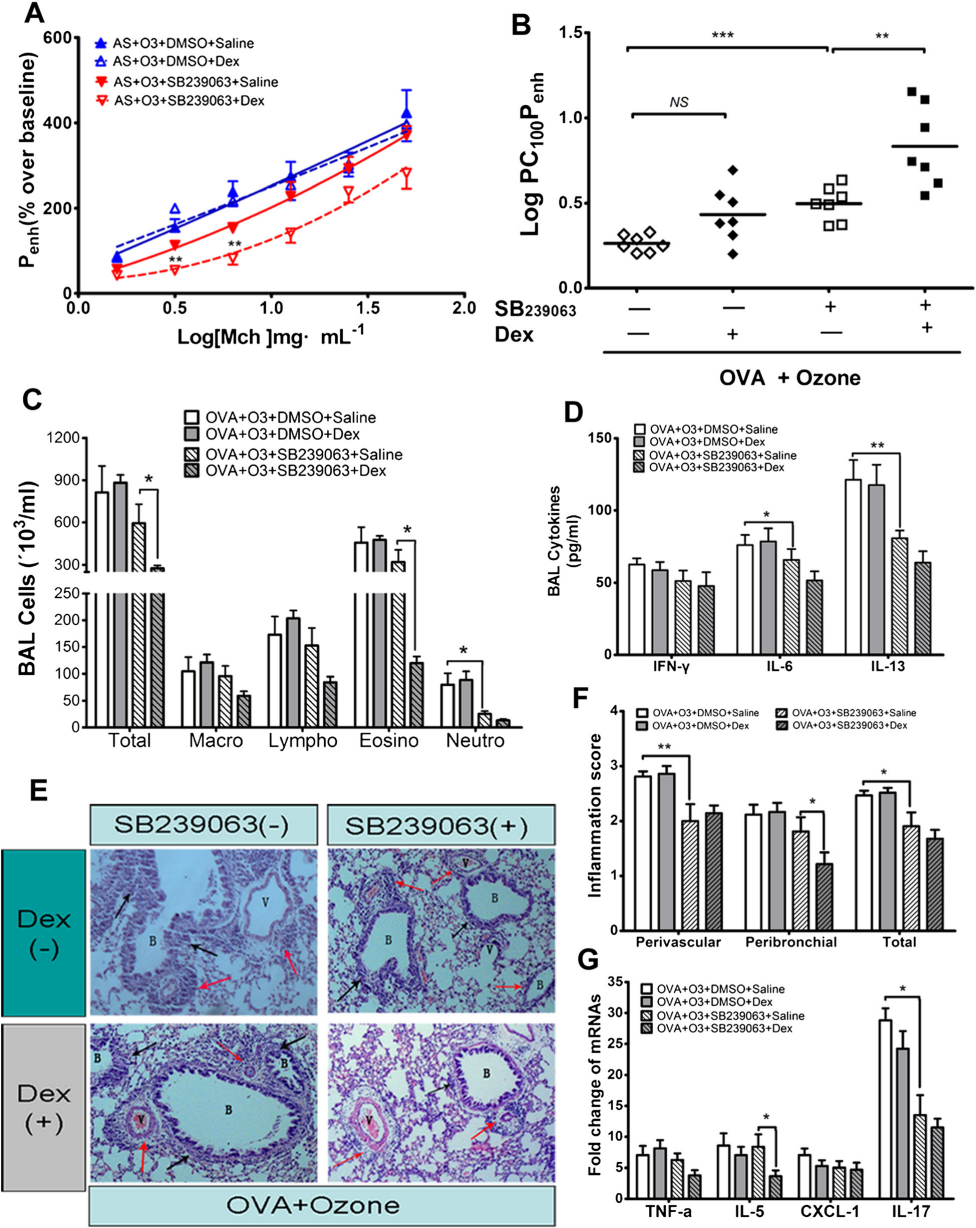
**SB239063 partly restored the ozone-impaired inhibitory effects of dexamethasone.** Twenty-eight asthmatic mice were exposed to 1.0 ppm ozone for 3 hrs. Then equal number of mice (14) received injection of SB239063(4.0 mg/kg, diluted by DMSO) or DMSO only 1 hr before and 4 hr after each O_3_ exposure. Half number of mice (7) in each group received intraperitoneal injection with dexamethasone (2.0 mg/kg) or saline only 1 hr before each aerosol challenge. Interpretation of **A** to **G** is same as Figure 2. *:p < 0.05; **:p < 0.01; ***: p < 0.001; n = 7.

Data showed that SB239063 in O_3_-exposed asthmatic mice significantly inhibited the AHR, decreased the number of BAL neutrophils and concentrations of IL-6 and IL-13, alleviated the perivascular lung inflammation and down-regulated the lung IL-17 mRNA expression. Furthermore, upon the delivery of SB239063 but not DMSO only, the administration of dexamethasone elicited some inhibitory effects on the AHR, the number of BAL total and eosinophil cells, the peribronchial lung inflammation, and the lung expression of IL-5 mRNA in O_3_-exposed asthmatic mice (Figure 4).

### SB239063-induced changes in oxidative stress, the p38 MAPK phosphorylation and the expression of MKP-1

**Figure 5:**
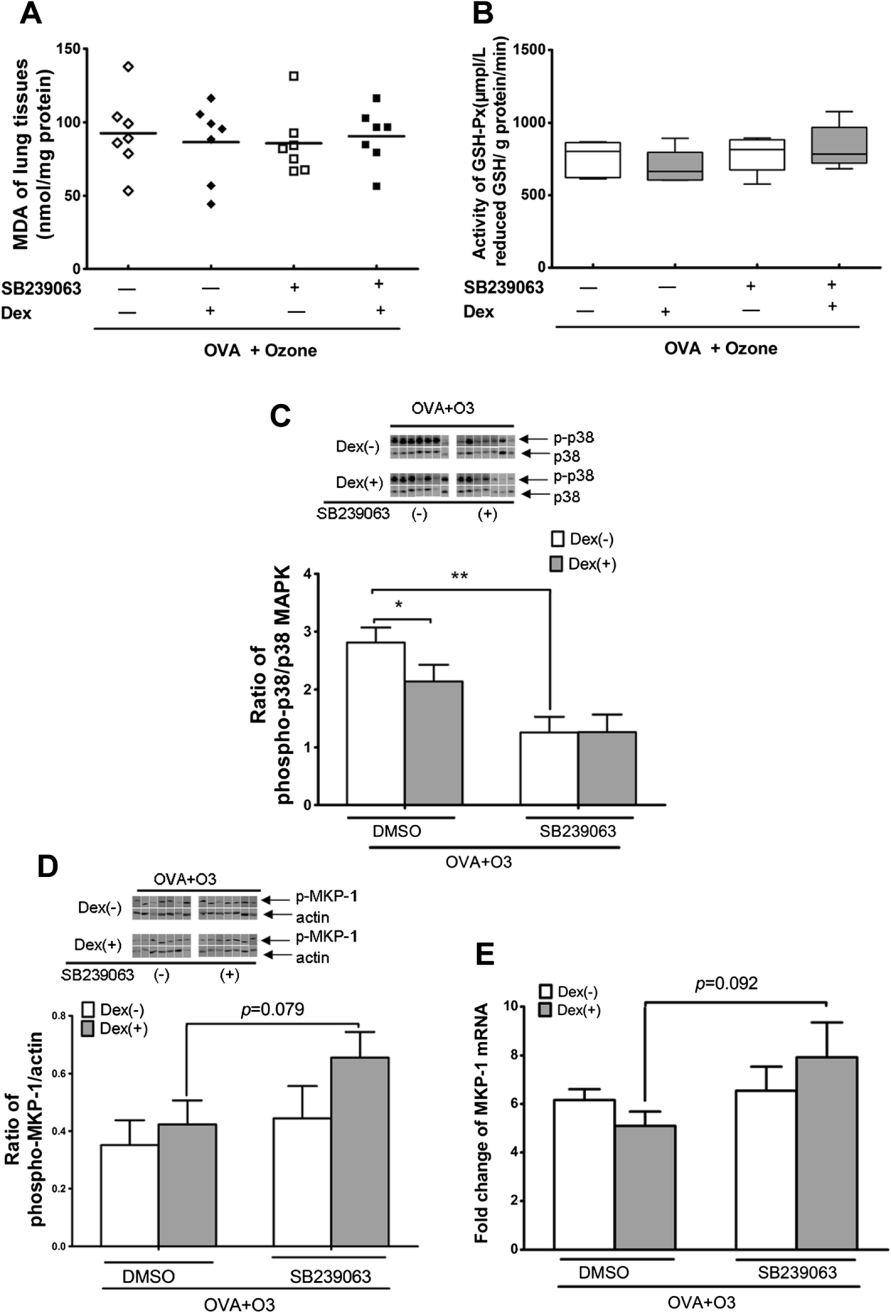
**SB239063 caused changes in oxidative stress and the phosphorylation of p38 MAPK and the expression of MKP-1 in lung tissues.** Interpretation of **A)** to **E)** is same to those in Figure 3. *:p < 0.05; **:p < 0.01; ***: p < 0.001; n = 7.

SB239063 affected neither the concentration of MDA nor the activity of GSH-Px in O_3_-exposed asthmatic mice (Figure 5A and B).

Compared with DMSO only, SB239063 significantly decreased the phosphorylation ratio of p38 MAPK, but not influenced the mRNA or the protein expression of MKP-1, in lung tissues of O_3_-exposed asthmatic mice (Figure 5C, D and E). However, upon the delivery of SB239063, further administration of dexamethasone caused a slight non-significant increase in the protein (SB239063_4mg/kg_: 0.656 ± 0.088 vs DMSO: 0.423 ± 0.084, *p* = 0.079) and mRNA (SB239063_4mg/kg_: 7.922 ± 1.427 vs DMSO: 5.093 ± 0.591, *p* = 0.092) expression of MKP-1, in O_3_-exposed asthmatic mice (Figure 5D and E).

## Discussion

As expected, the mice of the present allergic asthma model exhibited AHR, eosinophilic airway inflammation, and elevated BAL and lung immunological cytokines. Consistently with other reports, the enhanced phosphorylation of p38 MAPK was observed in the present animal model, which may indicate the participation of this kinase in the pathophysiological process of anaphylaxis [26,27]. Interestingly, the protein and gene expression levels of MKP-1 in the lungs of the experimental animals were also elevated. This phenomenon, which has not been reported heretofore, provides a clue that the p38 MAPK and MKP-1 might balance each other at a comparatively elevated baseline level within such an asthma model. Exposing these experimental animals to ozone enhanced the AHR, induced the neutrophilic airway inflammation, promoted the expressions of Th1 and Th17 cytokines in BAL and lung tissues, and increased the oxidative stress levels. These aggravating effects of ozone on the allergic animals have been demonstrated previously by other researchers and ourselves [12,28,29]. The activated p38 MAPK pathway is the cornerstone of the ozone-induced detrimental effects both on the AHR and airway inflammation in mice [7,15]. In the present study, we illustrated that the inhibition of p38 MAPK significantly eliminated the adverse activities of ozone on the AHR and airway inflammation as well as on most of the inflammatory cytokines, such as IL-6 and IL-13 in BAL, and IL-17 in the lung tissues of the asthmatic mice (Figure 4). It is reasonable to come to a conclusion that the p38 MAPK pathway plays central roles in the influence of ozone on current asthma model. However, the mRNA expressions of several inflammatory cytokines, such as CXCL-1 and TNFα, cannot be inhibited by SB239063, which may indicate that the expressions of these pro-inflammatory cytokines, whether being induced by allergic inflammation or by ozone stimulation, are regulated by an exceedingly complicated crosstalk network in which the p38 MAPK pathway is subtly involved. However, whether the oxidant ozone affects the therapeutic effects of corticosteroids on this allergic experimental model has not been reported yet.

The inhibitory effects of dexamethasone on the current experimental model were evidenced by the decreased AHR (increased LogPC100Penh), attenuated airway inflammatory cellularity and pathologic alleviation of the lung inflammation (peri-bronchial and peri-vascular area) as well as by the lowered mRNA expression of Th2 cytokines (IL-5) in the lungs. Interestingly, these dexamethasone-induced alterations were diminished in the mice of identical background airway inflammation but received the O_3_ exposure simultaneously with the medication. Accordingly, it can be modestly inferred that the subacute ozone exposure in the present study impaired the inhibitory effects of dexamethasone on this classical allergic asthma model. In other words, the ozone exposure blunted the response to the corticotherapy of these allergic mice.

The underlying mechanisms of the ozone-decreased biological response to glucocorticoids action remain unclear. However, we found in current study that the O_3_ exposure of the asthmatic mice caused four main specific alterations differing from their air-exposed counterparts. These particular features, namely the airway neutrophilia, enhanced activation of p38 MAPK, upregulated expression of IL-17, and altered expression of MKP-1, may lead to the resistance to the subsequent delivery of dexamethasone.

Neutrophils are generally considered less responsive to GCs compared to other inflammatory cells [30]. The neutrophilic inflammation existing in the context of some chronic conditions, such as the chronic obstructive pulmonary disease (COPD) and chronic asthma, tends to be resistant to corticotherapy, whether in human patients [10,31] or in experimental animals [32]. However, it does not mean that neutrophils are resistant to GCs by nature, for Hirsch et al. reported that neutrophils are as sensitive as other leukocytes to the inhibitory effects of glucocorticoids on the expressions of pro-inflammatory genes in peripheral blood mononuclear cells (PBMCs) of normal subjects [33]. Since the current study did not measure the direct response of neutrophils to the application of glucocorticoids, our data cannot offer further supporting information on whether a subacute oxidative stress conditions, as created in the present investigation, might render the accumulated neutrophils resistant to corticosteroids. However, some neutrophil-related cytokines, such as TNF-α, were proved to be insensitive to dexamethasone, as shown in this examination (Figure 2G). It has been previously reported by Onda et al. that TNF-α can reduce the GC-induced transactivation of endogenous genes as well as that of a reporter plasmid which contains a GC responsive element (GRE) in cultured human epidermal keratinocyte cells [34]. Furthermore, Van et al. demonstrated that TNF-α not only impaired the GR-dependent gene expression, but also downregulated the levels of both GR mRNA and protein [35]. Given the intimate relationship between TNF-α and neutrophils, it is reasonable to infer that the contribution of neutrophils to the GC insensitivity may involve the function of TNF-α.

In addition to boosting airway neutrophils, ozone exposure on present asthma model caused significant activation of p38 MAPK, a kinase involved in the pathogenesis of both the allergic and the ozone-induced airway inflammation and AHR [15,26]. In this study, we demonstrated that the inhibition of p38 MAPK not only mitigated the aggravating effects of O_3_ on pulmonary inflammation and AHR, but also restored partly the O_3_-impaired inhibitory effects of dexamethasone. Although numerous examinations have indicated that the inhibition of p38 MAPK can promote the cellular response to corticosteroids in vitro [36-38], demonstrating the roles of this kinase in the pathogenesis of glucocorticoids insensitivity, the current investigation confirmed this viewpoint in vivo. The phosphorylation of GRα by the activated p38 MAPK in PBMCs is reported to contribute to the glucocorticoids insensitivity of severe asthmatic patients [39]. Besides, p38 MPAK might also exert its roles through its downstream regulatory molecules, such as IL-17.

As reported previously, the expression of IL-17 mRNA can be upregulated by ozone exposure [17,40,41]. However, it is by far not known how ozone initiates the upregulation of this Th17 cytokine. The results of the present research showed that the O_3_-elevated IL-17 mRNA expression was decreased by the SB239063, suggesting the critical functions of the activated p38 MAPK executed in the course between the O_3_ exposure and IL-17 expression. Besides, the pivotal roles of p38 MAPK in the synthesis of IL-17 by CD4 (+) cells have been validated by previous studies [42,43]. Overall, we have reasons to infer that the p38 MAPK pathway might contribute to the ozone-induced upregulation of IL-17. This hypothesis is worthwhile and needs to be further substantiated in vitro.

Th17 cells and cytokines play pivotal roles in the ozone-induced airway hyperresponsiveness in mice [17]. Interestingly, the AHR of mice, induced by the transfer of Th17 cytokines or Th17-cells, is susceptible to becoming insensitive to the inhibition effects of dexamethasone [44]. In the current examination, the elevated expression of IL-17 mRNA was associated with the decreased response to dexamethasone in the ozone-exposed asthmatic mice and vice versa after they received the administration of BS239063. Actually, the contribution of Th17 cytokines to the pathogenesis of developing absence of sensitivity to glucocorticoids has already been confirmed by numerous ex vivo investigations [44-46]. Based on these established contributions of IL-17 to GC insensitivity, it may be hypothesized that O_3_ exposure of asthmatic mice may promote the production of IL-17 via the activation of p38 MAPK pathway, and hence it may impair the therapeutic effect of dexamethasone.

It is well known that MKP-1 mediates the anti-inflammatory effects of GCs [4,47-49]. Impairments in the expression of this phosphatase will compromise the action of GCs. In the present study, we measured the phosphorylated MKP-1 protein instead of the total protein, considering the fact that after its synthesis, the MKP-1 protein is quickly degraded and hence is transient, while the phosphorylation by ERK1/2 on its serine residues, Serine 359 and Serine 364, promotes its stability and hence possesses phosphatase activity [23]. Moreover, given the common option that extracellular regulated protein kinases (ERK) 1/2 is involved to a lower extent in the response to extrinsic stress, it is reasonable to assume that the ozone exposure has an insignificant impact on the phosphorylation of the MKP-1 protein. Hence, the p-MKP-1 protein measured in the current investigation can be regarded as the response of the change in the total MKP-1 protein rather than the variation of the phosphorylation intensity.

Our results demonstrated that GCs induced the mRNA and protein expression of MKP-1, which is consistent with previous published data [49]. According to the conclusions originating from an earlier examination [9], this induction progress is initiated by the intracellular ROS, of which this course is vulnerable to the outer source of oxidants. In our study, we observed that the ozone exposure diminished the favorable influence of dexamethasone on mRNA and protein expression of MKP-1 in asthmatic mice. This trend was accompanied by the ozone-impaired GC therapeutic effects. Furthermore, the blockade of p38 MAPK by SB239063 not only restored a part of the curative actions of Dex, but also regained the slight conducive trend of GCs on the expression of MKP-1. These observations provide inferences that the impaired GCs induction of MKP-1 expression might contribute to the ozone-induced GC insensitivity.

Numerous studies have placed their focus on the influence of oxidative stress on the expression of MKP-1, mostly in vitro xpeiments [50,51]. However, the manner in which the oxidative stress influences the expression of MKP-1 in vivo still remains elusive. In the present experiments, we observed that the O_3_ exposure in allergic mice caused higher expression of MKP-1 at the mRNA level, but, unexpectedly, not at the protein level. Although previous research illustrated that p38 MAPK mediates the oxidative stress-induced MKP-1 mRNA expression in vitro [50], we demonstrated that the inhibition of p38 MAPK did not prevent the transcriptional induction of MKP-1 mRNA by O_3_ in vivo. The substantiate role of p38 MAPK in the ozone induction of MKP-1 needs to be further explored in different systems with diverse stimuli. However, this is the first report, which highlights the ozone-induced discrepant alterations between the mRNA and the protein expression of MKP-1. The biological basis for this discrepancy is by far not clear. According to Kuwano et al., the H_2_O_2_ treatment promotes the translation of MKP-1 by increasing the association of the MKP-1 mRNA with its binding protein HuR and NF 90 in cultured HeLa cells [52]. However, the oxidative modification of MKP-1 protein, promoted by oxidative stress, not only inactivates MKP-1 but also targets this phosphatase for proteasomal degradation [53]. Furthermore, though the proper intracellular activation of ERKs contributes to the phosphorylation and the stability of the MKP-1 protein, the sustained activation of ERKs causes the degradation of MKP-1 protein via the ubiquitin-proteasome pathway [54]. Overall, we defined a hypothesis that this differential influence on the MKP-1 protein may result from the O_3_-enhanced oxidative stress-related post-translational modification, which needs to be further substantiated.

## Conclusions

In summary, present study demonstrated that ozone inhalation impaired the therapeutic effects of dexamethasone on AHR and airway inflammation of mice with allergic airway disease, which can be reversed by the inhibition of p38 MAPK. The pathophysiological basis underlying the ozone-induced declining sensitivity to GCs may involve the over phosphorylation of p38 MAPK, which may further mediate the upregulated IL-17 expression, and the elevated oxidative stress, which may disturb the GCs induction of MKP-1 expression and promote the degradation of MKP-1 protein. This study put forward an alternative explanation for the ozone oxidative stress induced insensitivity to glucocorticoids therapy, and provide the identification of new therapeutic targets which may aid in the management of patients with steroids-resistant asthma.

## Additional file

Additional file 1: Figure S1. Preliminary experiment for SB239063 dose titration.
