# Supplementary material for: Impact of ozone exposure on the response to glucocorticoid in a mouse model of asthma: involvements of p38 MAPK and MKP-1
Source: Respir Res. 2014 Oct 8;15(1):126. doi: 10.1186/s12931-014-0126-x (PMC4196074; doi:10.1186/s12931-014-0126-x)
Supplement: Additional file 1: Figure S1. — Preliminary experiment for SB239063 dose titration. [file 12931_2014_126_MOESM1_ESM.pdf]

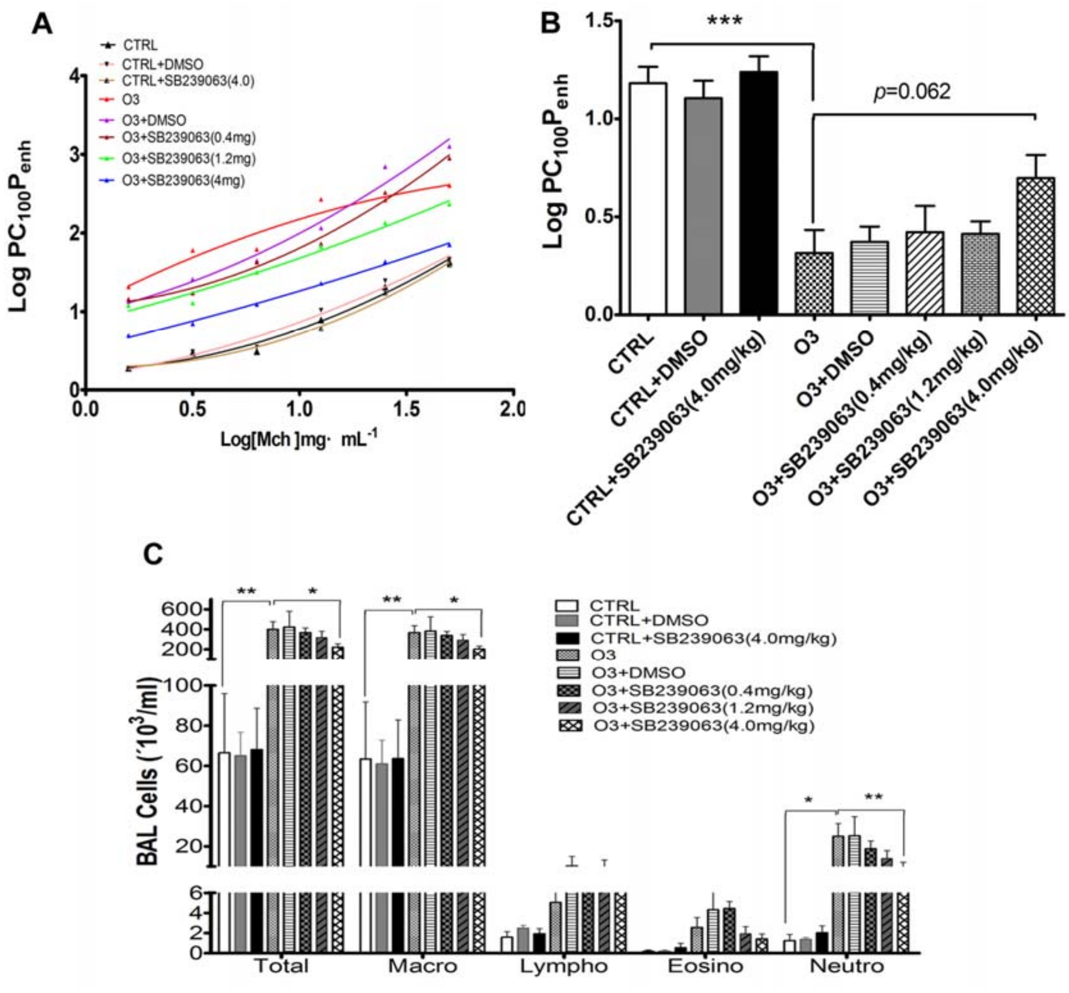

Figure S. **Preliminary experiment for SB239063 dose titration.** Mice were exposed to either 1.0 ppm ozone (O3) or filtered air (Ctrl) for three hours, in three every other days. Mice in different group (n=4) received different dose of SB239063(0.4mg/kg; 1.2mg/kg; 4.0mg/kg) or DMSO only, respectively. SB239063 were administrated by injection through tail vein 1hr before and 4hrs after O3 exposure. 24 hrs after the last exposure mice were measured for AHR and BAL cells. A: mean percentage increase in Penh to increasing provocative concentrations of Mch. B: log provocative concentration of MCh required to increase Penh from baseline to 100% (PC_{100}). C: changes in total cells and differential cellularity of BAL. *:p<0.05; **:p<0.01; ***: p<0.001; n=4
